# Contribution of Water from Food and Fluids to Total Water Intake: Analysis of a French and UK Population Surveys

**DOI:** 10.3390/nu8100630

**Published:** 2016-10-14

**Authors:** Isabelle Guelinckx, Gabriel Tavoularis, Jürgen König, Clémentine Morin, Hakam Gharbi, Joan Gandy

**Affiliations:** 1 Hydration and Health Department, Danone Research, Palaiseau 91767, France; isabelle.guelinckx@danone.com (I.G.); clementine.morin@danone.com (C.M.); hakam.gharbi@danone.com (H.G.); 2 CREDOC (Centre de Recherche pour l’Etude et l’Observation des Conditions de Vie), Paris 75013, France; tavoularis@credoc.fr; 3 Department of Nutritional Sciences, Faculty of Life Sciences, University of Vienna, Vienna 1090, Austria; juergen.koenig@univie.ac.at; 4 British Dietetic Association, Birmingham B3 3HT, UK; 5 School of Life and Medical Services, University of Hertfordshire, Hatfield AL10 9EU, UK

**Keywords:** total water intake, fluid intake, food moisture, adequate intake

## Abstract

Little has been published on the contribution of food moisture (FM) to total water intake (TWI); therefore, the European Food Safety Authority assumed FM to contribute 20%–30% to TWI. The aim of the present analysis was to estimate and compare TWI, the percentage of water from FM and from fluids in population samples of France and UK. Data from 2 national nutrition surveys (Enquête Comportements et Consommations Alimentaires en France (CCAF) 2013 and the National Diet and Nutrition Survey (NDNS) 2008/2009–2011/2012) were analyzed for TWI and the contribution of water from FM and fluids. Children and adults TWI were significantly lower in France than in the UK. The contribution of water from foods was lower in the UK than in France (27% vs. 36%). As TWI increased, the proportion of water from fluids increased, suggesting that low drinkers did not compensate by increasing intake of water-rich foods. In addition, 80%–90% of the variance in TWI was explained by differences in water intake from fluids. More data on the contribution of FM to TWI is needed to develop more robust dietary recommendations on TWI and guidance on fluid intake for the general public.

## 1. Introduction

With increasing recognition of the relationship and understanding of the mechanism between water and beverage intake, hydration and health [1,2,3,4], it is important to develop evidence-based recommendations and guidelines for water intake. Recommendations, such as those published by the European Food Safety Authority (EFSA) on adequate intakes (AIs) of total water intake (TWI) [5], form the basis of public health strategies, intervention programs and food based dietary guidelines. Recommendations on the AI of total water are based partly (e.g., EFSA) [5], or wholly on population survey intake data (e.g., Institute of Medicine (IOM)) [6]. It is important to emphasize that these recommendations are for TWI, i.e., water from fluids (drinking water and water from all other beverages) and water from foods (food moisture (FM)).

The water content of foods is highly variable. For example, cucumber and lettuce are approximately 96% water, whole, boiled chicken eggs are 75% water, while digestive (semi-sweet) biscuits contain only 2.8% water [7,8,9,10]. Clearly, the type and quantities of foods eaten will determine the contribution of FM in the overall diet to total water intake. Many factors influence the selection of foods including food availability, climate, cultural factors, health and economical status, age, psychosocial factors, religious factors and agricultural practices [11]. As a consequence, the amount of water obtained from food will vary between individuals and countries. For example, in China, food contributes 40% of TWI [12] while the IOM in USA estimated, when making its recommendations on AI of TWI, that, in adults, food contributed only 19% [6]. This was confirmed by a later analysis of the National Health and Nutrition Examination Surveys (NHANES) that estimated FM to be 19% in adults (NHANES 1999–2006) [13]. More recently, analyses of NHANES 2005–2010 have estimated FM to be 17%–25% in adults and 25%–30% in children aged 4–13 years [14,15]. In Mexico, FM has been estimated to be higher at 34.5% in children and adolescents aged 1–18 years [16] despite high intake of beverages by children in Mexico [17].

While data on the water content of foods are usually available in food composition tables and databases, the overall contribution of water in foods to TWI is seldom reported. In a recent survey of population nutrition and diet surveys in Europe, only one country reported calculating FM [18]. As a consequence of this lack of data, the EFSA assumed that the contribution of FM to TWI was 20%–30% when formulating their recommendations on AI of total water.

Dietary recommendations must be easily understood by the general public enabling them to know what they should eat and/or drink. Dietary reference values, such as EFSA’s scientific opinion on TWI, need to be put into a clear and readily understandable format such as food based dietary guidelines (FBDG), e.g., the German three-dimensional food pyramid [19], or the UK’s Eatwell Guide [20]. For TWI, it has to be considered that estimating water from food is difficult, if not impossible, for members of the general public. Therefore, FBDG should specify quantities of fluids (water and other beverages). Publications reporting on the mean water content of the population’s food would facilitate more accurate FBDG on water intake. However, not only the mean but also the variability of the ratio of water from FM to water from fluid across the population should be analyzed in order to identify those food and fluids promoting adherence to the AI of water. It is possible that foods rich in FM may ensure adherence to AI of TWI. In addition, publications of observed intake of TWI would benefit the development of future recommendations.

Therefore, the aim of the present analysis was to estimate and compare TWI and the contribution of water from FM and fluids to TWI in two population surveys (France and UK). In addition, it aimed to compare the ratio of water from FM and Fluids according to deciles of TWI and adherence to EFSA AIs for TWI.

## 2. Materials and Methods

Data from two nationally representative population surveys were accessed and analyzed; the French nutrition survey (called Enquête Comportements et Consommations Alimentaires en France (CCAF) 2013 translated as the Survey of Nutritional Behavior and Intakes in France) and the UK’s National Diet and Nutrition Survey (NDNS) 4 Year Rolling Programme 2008/2009–2011/2012.

### 2.1. The French Nutritional Survey (CCAF 2013)

The French survey was a descriptive, cross-sectional study conducted in 2012–2013 by the Research Centre for the Study of Life Conditions (CREDOC). This is a non-profit government organization that studies living and lifestyle conditions in France and periodically collects data about intake of food, energy, macro- and micronutrients of the French population.

The details of participant recruitment are consistent with the CREDOC methodology [21]. A quota method, taking into account age, socioeconomic level, region, town size and household size was used to recruit, through face-to-face interviews, a nationally representative sample of 2000 French households. The final sample for this analysis consisted of 901 children aged 3–18 years and 1062 adults aged 19 years and over. The data collection was conducted between October 2012 and July 2013. To control for seasonal differences in intake, the study was carried out in four successive phases (October–December, January–March, April–mid-June, mid-June–July), during each of which approximately a quarter of the participants were included.

A seven-day diary was used to assess all dietary intake (fluids and solids). The participants received written instructions on how to complete the seven-day food and fluid diary. The diary was used to record information on all eating and drinking occasions throughout the day and for individual eating occasions. The circumstances of intake (time and location) were noted. Respondents completed the food diary with the aid of a validated photographic booklet [22], which presented various common foods and beverages in different portion sizes. Investigators ensured completeness of the seven-day diary through face-to-face interviews. The water content of reported intakes was obtained from a recognized French food composition table [23].

### 2.2. The UK Nutritional Survey (NDNS 2008/2009–2011/2012)

The NDNS is a survey of the health and diet of a nationally representative sample of adults and children in the UK. The present survey was funded by Public Health England (Department of Health) and the Food Standards Agency and conducted by NatCen Social Research, MRC Human Nutrition Research (HNR) and the University College London Medical School. It was conducted as a 4 year rolling programme from 2008/2009 to 2011/2012 [24] and all seasons were sampled.

One adult (aged 19 years and over) and one child (aged 1.5–18 years) were randomly selected from individual households. A sample was taken from a list of all addresses in the UK and clustered into Primary Sampling Units (PSUs) based on small geographical areas, which were randomly selected from postcode sectors. A sample of 21,573 addresses was then randomly selected from the PSUs, which had also been randomly selected. A total sample of 4156 individuals aged 1.5 and over (including 386 young children aged 1.5–2 years, 1687 children aged 3–18 years and 2083 adults aged 19 years and over) completed three or four dietary recording days for years 1–4 combined. To ensure comparability between age groups of both surveys, data from children aged less than four years was excluded in the NDNS sample. Some individuals later agreed to be interviewed by a nurse with fewer agreeing to give blood or urine samples. The data were weighted to minimize selection bias. All relevant research ethics and governance committees approved the survey.

Subjects were asked to complete a four-day estimated food diary with a randomly selected start date, which facilitated sampling across all days of the week. An interviewer visited the household to explain how to keep the four-day food diary. Interviewers visited or phoned the household on day two or three to check the ongoing diary completion and visited to review and collect the diary no more than three days after the final recording day. Photograph atlases were provided to aid estimation of portion sizes. Parents or carers assisted children aged 11 years and younger with completion of the diary. A trained nurse measured height and weight on a subsequent visit.

Completed diaries were coded and entered into HNR’s dietary assessment system (DINO, Diet In Nutrients Out) that used food composition data from the Department of Health’s NDNS Nutrient Database to estimate dietary intake of foods, including water and beverages and nutrients, including energy and water. Computerized raw data files and documentation from this survey were obtained under license from the UK Data Archive [25].

The food groups considered as fluids are shown in supplementary materials Table S1. Soup was categorized as a food and milk as fluid.

### 2.3. Statistical Analysis

The SAS 9.2 software (SAS Institute Inc., Cary, NY, USA) was used for statistical analysis. The data of TWI were normally distributed. Continuous variables are presented as mean and standard deviation as appropriate and dichotomous variables as frequency and percentage. The number and proportion of participants with an inadequate TWI was calculated by comparing TWI to the age and gender specific AI of TWI set by EFSA [5], which are shown in Table 1. General linear models were used to identify the variation in TWI due to water from fluids or FM.

Before analyzing the four waves of NDNS as one sample, a statistical comparison between the waves was performed. A significant difference in TWI between waves was observed only among subjects aged 11–18 years (*p* = 0.006), and not among subjects aged 4–10 years, 19–64 years and ≥65 years. Since the difference in TWI between consecutive waves was on average 63 mL/day and not consistent across age groups, aggregating the four waves for analysis was considered appropriate.

Students’ *t*-test was used for comparative statistics; a *p*-value < 0.05 was considered significant. These data were normally distributed and are reported as mean (SD). Data of water intake were not normally distributed and are reported as median (interquartile ranges (IQR)).

## 3. Results

There were significant differences in demographic data for the two countries as shown in Table 2. French children were significantly lighter (*p* = 0.002 and 0.001 for males and females, respectively); however, French children had significantly higher energy intakes (*p* = 0.0007 and < 0.0001 for males and females respectively). Similarly, UK adults were significantly heavier than French adults despite reporting significantly lower energy intakes.

### 3.1. Contribution of Water from Food Moisture and Fluids to Total Water Intake According to Country, Gender and Age Groups

TWI was significantly higher in males compared with females in France (*p* = 0.0055) and the UK (*p* < 0.0001) as shown in Table 3. Mean TWI in French children was significantly lower than UK children (*p* = 0.019). The contribution of water from fluids was consistently higher in the UK than in France, overall (73% vs. 64%) and for each age and gender category. In France, males had a higher proportion of water coming from fluids; however, in the UK, there was little difference between the genders for all age groups. Water intake from fluids and food moisture were significantly higher in UK adults than in French adults (both *p* < 0.0001).

### 3.2. Contribution of Water from Food Moisture and Fluids to TWI According to Deciles of Total Water Intake

**Figure 1:**
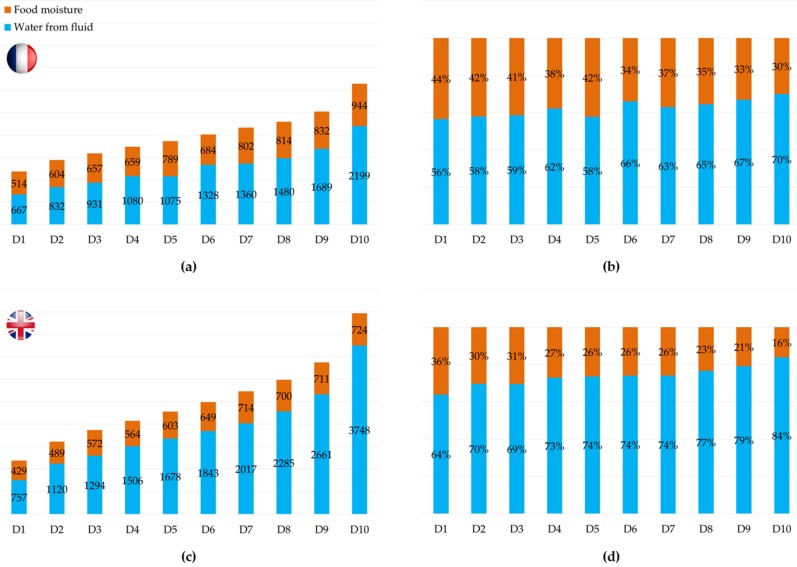
Volume and contribution to total water intake of water from fluid and food moisture in the male adult sample of the French 2013 survey called Enquête Comportements et Consommations Alimentaires en France (CCAF) (**a**,**b**) and the UK surveys called National Diet and Nutrition Survey (NDNS) 2008/2009–2011/2012 (**c**,**d**).

Figure 1 shows TWI and the proportion of water from food and fluids split into deciles for male adults. In both countries, as TWI increased, the proportion of water from fluids increased accordingly. The finding was consistent in the female adults (supplementary materials Figure S1) and children (supplementary materials Figure S2).

**Figure 2:**
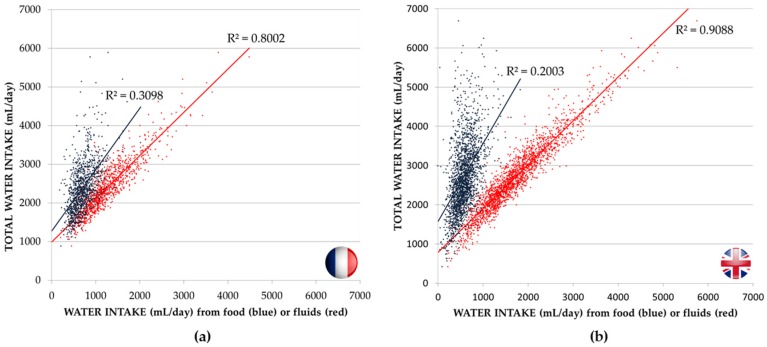
Water from food moisture (in **blue**) or total fluid intake (in **red**) as a function of total water intake in the adult sample of the French CCAF 2013 survey (**a**) and the UK NDNS 2008/2009–2011/2012 surveys (**b**).

Figure 2 shows the relationship between TWI and water from food or fluids in adults (male and female) in France (a) and the UK (b). There was a higher correlation between TWI and water from fluids than between TWI and water from FM for adults in France (*R*^2^ = 0.8002 vs. *R*^2^ = 0.3098) and the UK (*R*^2^ = 0.9088 vs. *R*^2^ = 0.2203). These regressions suggested that 80%–90% of the variance in TWI was explained by differences in water intake from fluids. In children, the variance in TWI was explained by differences in water from fluids and to a lesser extent with water from foods (*R*^2^ = 0.7612 vs. *R*^2^ = 0.4766 in France and *R*^2^ = 0.8868 vs. *R*^2^ = 0.2699 in the UK; supplementary materials Figure S3).

### 3.3. Contribution of Water from Food Moisture and Fluids to TWI According to Adherence to EFSA Adequate Intake

In both countries, most children (88%–90%) had TWI below the EFSA recommendations for AI (Table 4). Fewer adults achieved the EFSA recommendations in France than in the UK; women were consistently more likely to have adequate intakes than men. In France, those who achieved the recommendations drank more than those who did not: children drank about 629 mL more and adults 804–884 mL more, representing a 6% to 7% higher contribution of water from fluids than from food. In the UK, there was a mean difference of 920–986 mL in water from fluids between those who achieved the recommendations and those who did not. This represented a 10% higher contribution of water from fluids to TWI than from food.

## 4. Discussion

Given the increasing interest in water intake and health, it is important that population studies be designed and executed with due consideration of the methodological issues inherent in recording fluids. In addition to reporting, detailed information on TWI and its sources is essential. Therefore, this study aimed to analyze and report the contribution of water from fluids and food moisture to TWI in two nationally representative surveys. Independently of gender, adults (≥19 years) in the French survey had lower TWI intakes compared with UK adults. This finding is mirrored in studies of total fluid intake (TFI) with French adults having lower intakes compared with those in the UK [26]. The TWI observed in the 2008/2009–2011/2012 NDNS is comparable to the analysis of the 2002/2001 NDNS data, which showed that adult men had a TWI of 2.53 ± 0.86 L/day and women a TWI of 2.03 ± 0.71 [27]. The TWI of children was comparable to analyses of other TFI data sets that also showed lower TFI in French children compared with the UK [28]. The TWI for French children was comparable to data from another French national survey (INCA) conducted in 2006/2007 [29]. Data on TWI in children in the UK from other analyses have not been published to date.

Regardless of the difference in TWI between both surveys, many children and adults in both countries had TWI below the EFSA (2010) AIs. This was especially true for children, nearly 90% of whom had a TWI lower than the AI. However, the median intakes for UK adults were close to the recommendations. The French results are similar to those of Vieux et al. who reported that 89%–93% in children aged 4–13 years had intakes less than EFSA’s AIs [29]. No comparable studies are available on TWI for the UK, although Iglesia et al. [28] and Gandy [30] have reported that 30%–56% of children and adolescents did not achieve the AIs when estimating TFI alone. Despite these differences, the present study and previous analyses show that the observed median intake of the sample is lower than the age- and gender-specific AI. However, when making such comparisons, it is important to consider the purpose of the AIs and how they were developed, and to only draw generalizable conclusions. The AIs are partly based on population intakes and are designed for population comparisons not for use in individuals. It is also important to note that, in the absence of hydration biomarkers, an intake below AI does not automatically equate to underhydration or dehydration. However, there is increasing evidence that, in children, dehydration and an increased fluid intake may impact cognitive and physical performance, and overall well-being [31,32,33]. Therefore, it would seem prudent to include biomarkers in future surveys in order to establish if those with reported low TWIs are at risk of mental and/or health issues.

There are undoubtedly many reasons for the differences in TWI between French and UK adults including climate, physical activity, assessment method and psychosocial factors. It is doubtful that these differences could be explained by climate, as while Northern France has a climate similar to the UK, the south is generally hotter than the UK. Likewise, since activity levels are low in both samples, they are unlikely to be explained by differences in physical activity levels. In addition, differences in methodologies may have contributed to these differences, even though both surveys used food diaries. Social factors are likely to explain some, if not most, of the variation. Dietary intake patterns vary between the countries. For example, Bellisle et al. estimated that, in France, 80%–87% of TFI was drunk *during* meals [34], and similarly Vieux et al. estimated that nearly 70% of all fluids are being consumed during three principle meals [29]. In the UK, the opposite was observed in a fluid specific survey; up to 70% of beverages were consumed *outside* of meals [30], when there are obviously more opportunities to consume fluids.

The importance of fluid intake to obtain a higher TWI was confirmed in the present analysis when looking at the contribution of FM to TWI. Indeed, the regression analyses demonstrated that differences in TWI were predominately due to differences in the amount of water from fluids. This was confirmed by the analysis of the ratio of FM and water from fluids by deciles of TWI; as the contribution of water from fluids to TWI increased 13%–20% between the first and tenth deciles in male adults in France and UK, respectively. However, the volume of this increase appeared more important (e.g., difference in male adults 1532 mL in France and 2991 mL in the UK). This analysis also suggests that participants with a low water intake from fluids did not compensate by having a higher intake of FM.

The mean percentage contribution of water from FM to TWI in the UK sample was consistently lower due to more fluids being consumed than among the French sample—27% and 36%, respectively. Similar values have been reported from earlier surveys in France: using data from the 2002/2003 CCAF survey Bellisle et al. estimated FM to be 36%–38% in French children and 36%–41% in French adults [34]. Similarly, Vieux et al. estimated FM to be 40% of TWI in French children aged 4–13 years [29]. In the UK, no specific data on FM have been reported for children; however, values of 23%–32% for all age groups (1.5–64 years) [35] and 25% in adults [27] have been reported, values in line with those in the present analysis. In other European countries, FM levels of 33%–38% have been reported in German children [36] and 32% for 9–75 year olds in Spain [37]. O’Connor has reported a FM of 33% for adults in the Republic of Ireland [38]. Therefore, the current analysis and the existing literature seem to confirm the EFSA’s assumption that FM contributes for 20%–30% to TWI. However, this ratio may not be applicable to all European countries [5]. It would be beneficial if future national food surveys record and report both TWI and the contribution of water from FM and fluids. More primary data published on this topic will enable the development of robust dietary recommendations on TWI. Moreover, further exploration of the contribution of specific foods, especially high water content foods such as fruit and vegetables, to total FM may yield additional insight into these country differences, as would an exploration of social differences between countries regarding drinking and eating behavior.

The present analysis has several strengths. Both surveys used an estimated food diary, a method that is considered to be the most robust available dietary assessment method [39]. Using surveys with the same assessment method was a selection criterion since different methods could create a bias in the results. For example, fluids can be underestimated when intake is assess with a 24 h recall and, consequently, this could bias the ratio of water from FM/water from fluids [40]. The collection of data across all seasons in both surveys will have minimized seasonal variations in food choices e.g., variation in type and quality of fruit and vegetables or fluids consumed. Such variations would have influenced the findings in this study due to variations in FM.

However, some limitations in the present analysis must be acknowledged besides those inherent in dietary surveys. While both surveys are considered representative of the respective country’s populations, the sampling methods were slightly different. The data from the UK is a summary of four years of a rolling program as opposed to France’s one year survey, which is repeated on a three-year basis. This resulted in significantly different sample sizes, with over twice as many subjects being surveyed in the UK compared with France. Additionally, in France, a seven-day diary was used while a four-day diary was used in the UK. However the four-day diary used by NDNS was validated against the previously used seven-day diary, and, therefore, is considered as representative and comparable [41]. Any dietary survey is subject to potential errors inherent in the methodology, for example underreporting. In this analysis, energy intake was lower than in the UK than in France, despite UK children and adults being heavier. Interestingly, TWI and its sources were lower in France, clearly demonstrating the need for methodologies validated for specific nutrients.

## 5. Conclusions

The findings, together with the current literature, indicated that TWI and the contribution of water from FM and fluids were variable between countries. Also within one country, TWI as well as the contribution of water from FM varied greatly. More publications of primary data analysis from more countries on this topic are needed to develop more robust dietary recommendations on TWI and FBDG, preferably on fluid intake in addition to TWI. Water from fluids was shown to be the main driver of TWI. Encouraging the consumption of fluids, especially drinks that do not contribute to total energy intake such as water, seems therefor appropriate to increase TWI to the levels recommended by EFSA.

## Figures and Tables

**Table 1:** Dietary reference intakes (adequate intakes) for total water set by the European Food Safety Authority (EFSA) [5].

Age and Physiological Classes			Total Water Adequate Intake
Infants	0–6 months		680 mL/day through milk
	6–12 months		800–1000 mL/day
Children	1–2 years		1100–1200 mL/day
	2–3 years		1300 mL/day
	4–8 years		1600 mL/day
	9–13 years	Boys	2100 mL/day
		Girls	1900 mL/day
	>14 years		Same as adults
Adults	Men		2500 mL/day
	Women		2000 mL/day
Pregnant women			+300 mL/day vs. adults
Lactating women			+600–700 mL/day vs. adults
Elderly			Same as adults

**Table 2:** Demographics of the both survey samples.

	CCAF 2013		NDNS 2008/2009–2011/2012		*p*-Values	
	France		UK			
	Males	Females	Males	Females	Males	Females
4–18 years						
Sample size	478	423	859	828		
Weight (kg)	41 ± 17	41 ± 17	45 ± 13	44 ± 14	0.0002	0.0001
Height (m)	146 ± 23	144 ± 21	148 ± 15	145 ± 14	0.1407	0.5264
Energy Intake (kcal/day)	1906 ± 598	1636 ± 478	1802 ± 303	1540 ± 244	0.007	<0.0001
≥19 years						
Sample size	426	636	901	1182		
Weight (kg)	78 ± 14	65 ± 12	85 ± 20	72 ± 20	<0.0001	<0.0001
Height (m)	175 ± 8	163 ± 6	175 ± 10	161 ± 8	0.3374	0.0002
Energy Intake (kcal/day)	2229 ± 523	1832 ± 399	2109 ± 851	1588 ± 591	0.0018	<0.0001

Data are presented as mean ± standard deviation. Abbreviations: CCAF Enquête Comportements et Consommations Alimentaires en France, NDNS National Diet and Nutrition Survey.

**Table 3:** Contribution of water from food and fluids to total water intake (TWI) according to country, gender and age groups.

			TWI	Water from Fluids		Water from Food	
		*N*	(mL/Day) *	(mL/Day) *	% TWI	(mL/Day) *	% TWI
**CCAF 2013—France**							
4–10 years	Males	252	1254 (1074–1519)	738 (616–912)	61%	483 (405–606)	39%
	Females	194	1213 (1016–1519)	752 (585–948)	64%	450 (372–549)	36%
11–18 years	Males	226	1510 (1255–1818)	951 (740–1182)	62%	560 (473–689)	38%
	Females	229	1382 (1143–1649)	846 (676–1055)	64%	520 (394–617)	36%
19–64 years	Males	324	1922 (1559–2273)	1188 (921–1573)	65%	671 (538–814)	35%
	Females	492	1763 (1437–2143)	1139 (840–1458)	66%	597 (488–745)	34%
≥65 years	Males	102	1929 (1699–2325)	1115 (874–1420)	59%	810 (664–972)	41%
	Females	144	1921 (1633–2373)	1130 (882–1460)	61%	782 (638–923)	39%
4–18 years	total	901	1358 (1114–1645)	825 (650–1055)	65%	508 (402–611)	35%
≥19 years	Males	426	1923 (1591–2281)	1186 (916–1532)	62%	697 (569–858)	38%
≥19 years	Females	636	1796 (1479–2199)	1135 (847–1458)	65%	631 (508–795)	35%
**NDNS 2008/2009–2011/2012—UK**							
4–10 years	Males	414	1253 (1045–1509)	837 (664–1056)	67%	418 (343–502)	33%
	Females	389	1225 (1016–1470)	789 (627–994)	67%	411 (328–508)	33%
11–18 years	Males	445	1588 (1289–1978)	1110 (860–1486)	72%	463 (367–570)	28%
	Females	439	1348 (1083–1678)	934 (699–1249)	72%	392 (316–482)	28%
19–64 years	Males	710	2415 (1890–3018)	1793 (1306–2369)	76%	573 (457–732)	24%
	Females	945	2060 (1692–2578)	1522 (1150–1981)	75%	536 (413–656)	25%
≥65 years	Males	191	2260 (1796–2703)	1645 (1200–2026)	72%	628 (503–764)	28%
	Females	237	2002 (1692–2468)	1466 (1170–1840)	72%	567 (460–665)	28%
4–18 years	total	1687	1352 (1102–1686)	920 (692–121)	67%	416 (337–522)	33%
≥19 years	Males	901	2386 (1875–2968)	1745 (1292–2289)	76%	591 (469–739)	24%
≥19 years	Females	1182	2050 (1692–2559)	1510 (1153–1921)	72%	542 (425–658)	28%

* Data presented as median (25th–75th percentile); Abbreviations: CCAF Enquête Comportements et Consommations Alimentaires en France, NDNS National Diet and Nutrition Survey, TWI total water intake.

**Table 4:** Contribution of water from food moisture and water from fluids to total water intake of children (3–18 years), male and female adults (≥19 years) in France and UK according to intakes above or below the EFSA adequate intake (AI) of total water [5].

			TWI	Water from Fluids		Water from Food	
		*N* (%)	mL/Day *	mL/Day *	% TWI	mL/Day *	% TWI
**CCAF 2013—France**							
Children	<AI	811 (91%)	1310 (1082–1528)	786 (629–985)	62%	498 (398–593)	38%
	≥AI	85 (9%)	2079 (1849–2276)	1450 (1233–1641)	68%	627 (505–800)	32%
Male adults	<AI	435 (84%)	1832 (1534–2144)	1102 (879–1371)	62%	675 (546–810)	38%
	≥AI	82 (16%)	2752 (2609–3100)	1928 (1680–2195)	68%	870 (731–1093)	32%
Female adults	<AI	361 (64%)	1592 (1338–1776)	950 (754–1131)	61%	574 (473–714)	39%
	≥AI	199 (36%)	2384 (2167–2689)	1585 (1405–1964)	69%	778 (607–960)	31%
**NDNS 2008/2009–2011/2012—UK**							
Children	<AI	1477 (88%)	1288 (1056–1526)	864 (675–1093)	68%	404 (329–502)	32%
	≥AI	210 (12%)	2186 (1893–2665)	1688 (1349–2161)	78%	520 (405–637)	22%
Male adults	<AI	495 (56%)	1922 (1586–2204)	1326 (1089–1615)	71%	528 (422–658)	29%
	≥AI	406 (44%)	3086 (2794–3579)	2377 (2072–2883)	79%	682 (540–834)	21%
Female adults	<AI	538 (47%)	1661 (1396–1831)	1141 (907–1301)	69%	477 (374–580)	31%
	≥AI	644 (53%)	2493 (2227–2932)	1894 (1648–2312)	77%	606 (483–710)	23%

* Data presented as median (25th–75th percentile); Abbreviations: AI adequate intake, CCAF Enquête Comportements et Consommations Alimentaires en France, NDNS National Diet and Nutrition Survey, TWI total water intake.

## Supplementary Materials

The following are available online at http://www.mdpi.com/2072-6643/8/10/630/s1, Figure S1: Volume and contribution to total water intake of water from fluid and FM in the female adult sample of the French CCAF 2013 survey (a,b) and the UK NDNS (c,d) 2008/2009–2011/2012 surveys, Figure S2: Volume and contribution to total water intake of water from fluid and food moisture in the children’s sample of the French CCAF 2013 survey (a,b) and the UK NDNS (c,d) 2008/2009–2011/2012 surveys, Figure S3: Water from food moisture (in blue) or total fluid intake (in red) as a function of total water intake in the children’s sample (4–18 years) of the French CCAF 2013 survey (a) and the UK NDNS 2008/2009–2011/2012 surveys (b), Table S1: the categorization and codes of fluids used in analysis of the CCAF survey and NDNS survey.
