# Supplementary material for: Contribution of Water from Food and Fluids to Total Water Intake: Analysis of a French and UK Population Surveys
Source: Nutrients. 2016 Oct 14;8(10):630. doi: 10.3390/nu8100630 (PMC5084017; doi:10.3390/nu8100630)
Supplement: Supplementary file 1 [file nutrients-08-00630-s001.docx]

Supplementary Materials: Contribution of Water from Food and Fluids to Total Water Intake: Analysis of a French (CCAF) and an UK (NDNS) Population Survey

Isabelle Guelinckx, Gabriel Tavoularis, Jürgen König, Clémentine Morin, Hakam Gharbi and Joan Gandy

**Table S1.** Categorization and codes of fluids used in analysis of the CCAF survey and NDNS survey.

| **CCAF** | **NDNS** | | | |
| --- | --- | --- | --- | --- |
|  | **Code** | **Main Food Groups** | **Code** | **Sub Food Groups** |
| 4: Boissons alcoolisées  (alcoholic beverages) | 47 | Spirits and liqueurs |  |  |
|  | 48 | Wine |  |  |
|  | 49 | Beer lager cider & perry |  |  |
| 5: Boissons chaudes  (hot beverages) | 51 | Tea coffee and water | 51a | Coffee (made-up weight) |
|  |  |  | 51b | Tea (made-up weight) |
|  |  |  | 51c | Herbal tea (made-up weight) |
| 10: Eaux  (water) | 51 | Tea coffee and water | 51d | Bottled water still or carbonated |
|  |  |  | 51r | Tap water only |
| 16: Jus et nectars  (juices and nectars) | 45 | Fruit juice |  |  |
|  | 61 | Smoothies 100% fruit and/or juice |  |  |
| 17: Lait nature  (natural milk) | 10 | Whole milk |  |  |
|  | 11 | Semi skimmed milk |  |  |
|  | 12 | Skimmed milk |  |  |
|  | 13 | Other milk and cream | 13a | Infant formula |
|  |  |  | 13r | Other milk |
|  | 60 | 1% fat milk |  |  |
| 33: Sodas et autres BRSA  (soda’s and others) | 50 | Miscellaneous | 50a | Beverages dry weight |
|  |  |  | 50e | Nutrition powders and drinks |
|  | 52 | Commercial toddlers foods and drinks | 52a | Commercial toddlers drinks |
|  |  |  | 52r | Commercial toddlers foods |
|  | 57 | Soft drinks not low calorie |  |  |
|  | 58 | Soft drinks low calorie |  |  |


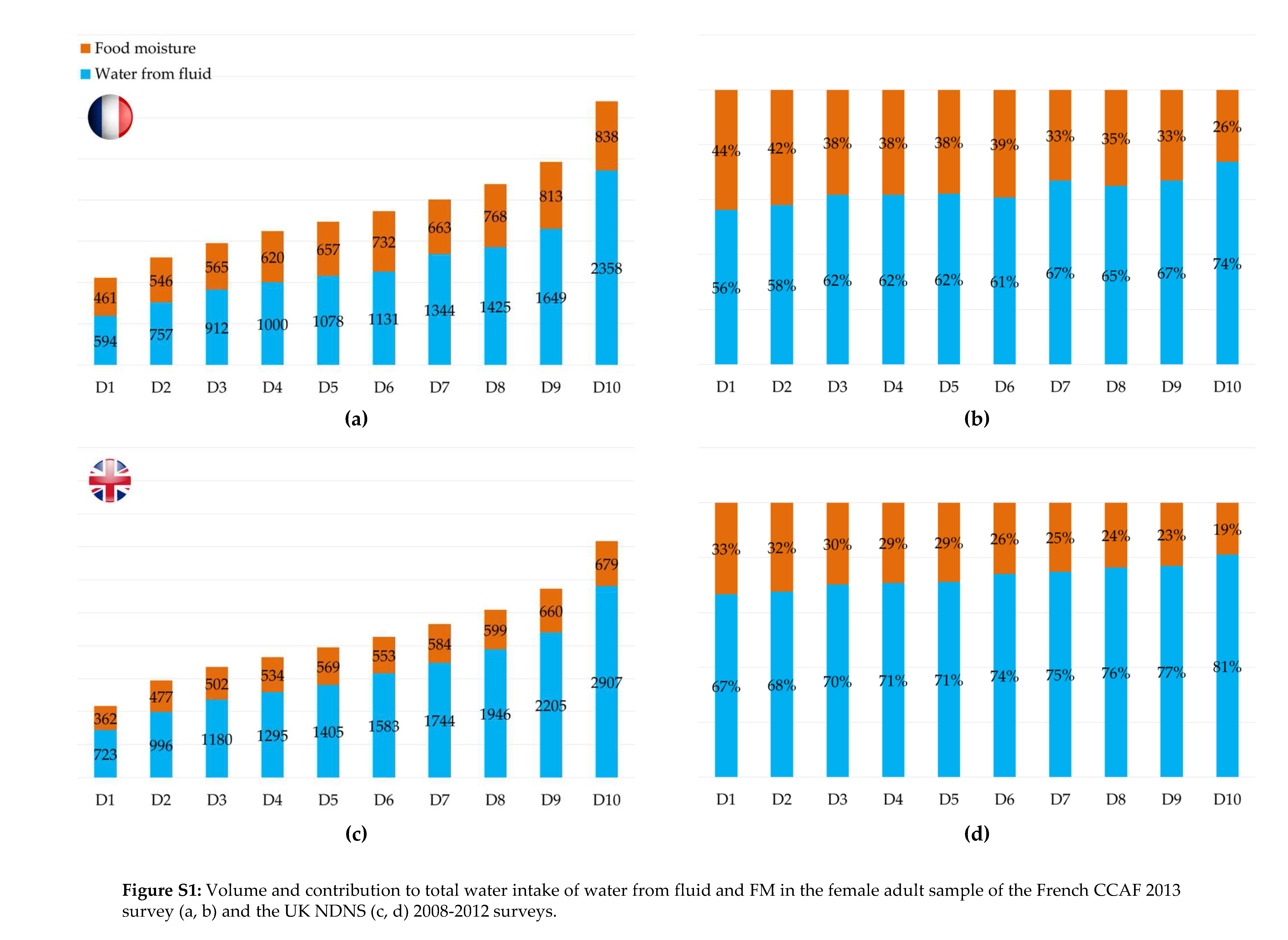


**Figure S1.** Volume and contribution to total water intake of water from fluid and FM in the female adult sample of the French CCAF 2013 survey (**a**,**b**) and the UK NDNS (**c**,**d**) 2008–2012 surveys.


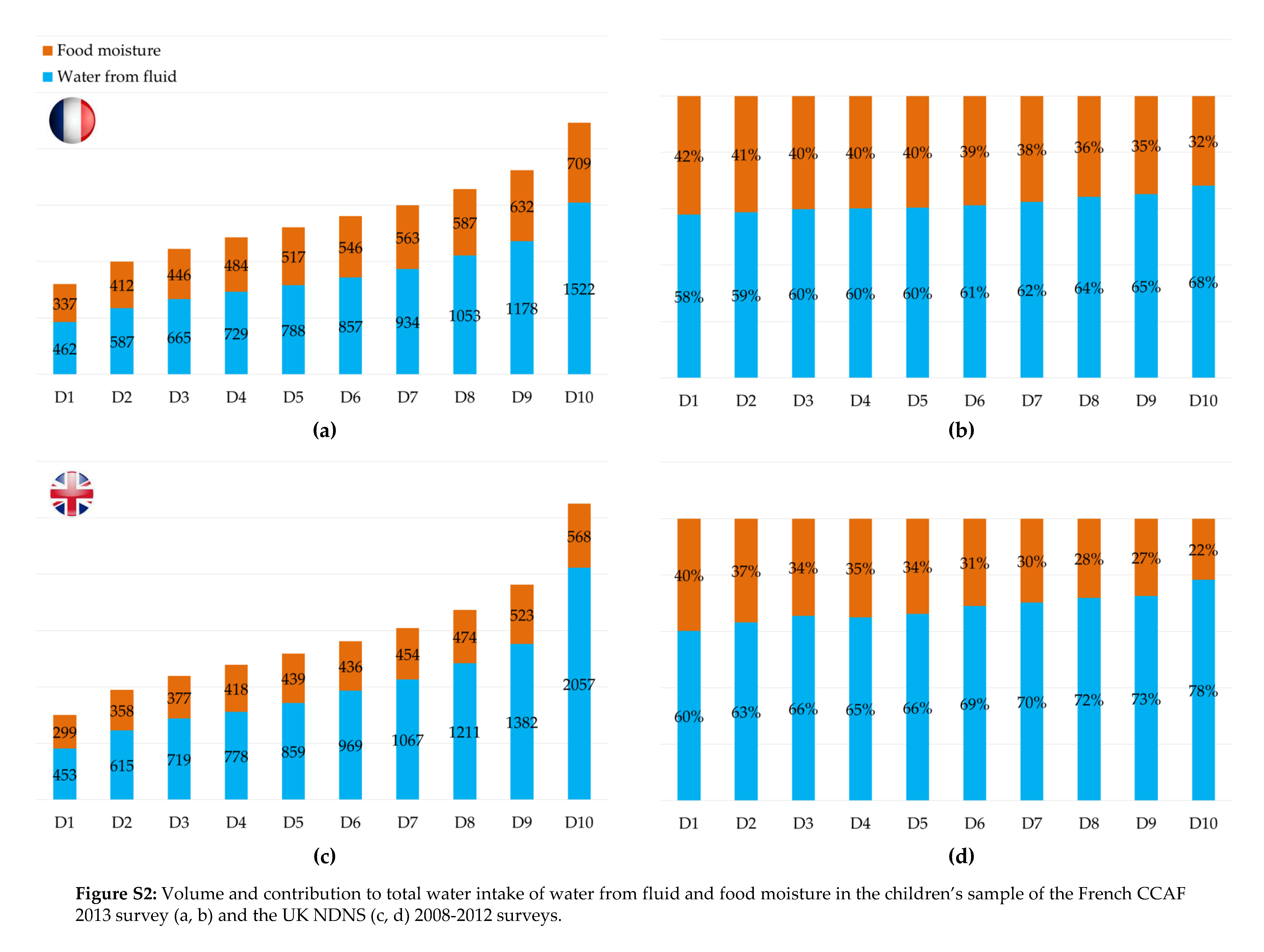


**Figure S2.** Volume and contribution to total water intake of water from fluid and food moisture in the children’s sample of the French CCAF 2013 survey (**a**,**b**) and the UK NDNS (**c**,**d**) 2008–2012 surveys.


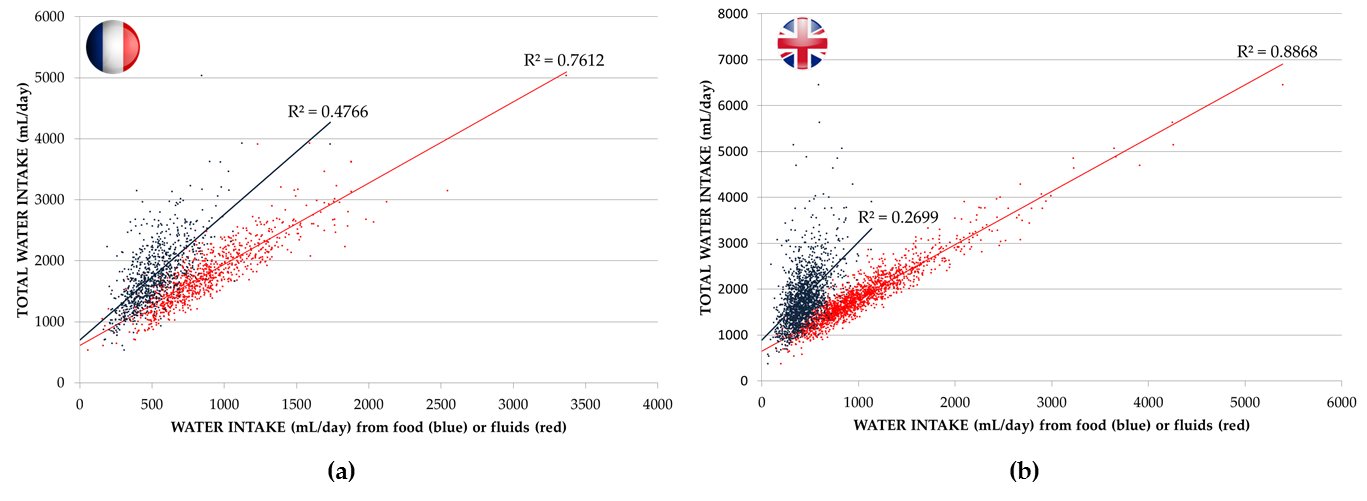


**Figure S3.** Water from food moisture (in blue) or total fluid intake (in red) as a function of total water intake in the children’s sample (4–18 years) of the French CCAF 2013 survey (**a**) and the UK NDNS 2008–2012 surveys (**b**).
